# Novel Antibacterial Copolymers Based on Quaternary Ammonium Urethane-Dimethacrylate Analogues and Triethylene Glycol Dimethacrylate

**DOI:** 10.3390/ijms23094954

**Published:** 2022-04-29

**Authors:** Marta W. Chrószcz, Izabela M. Barszczewska-Rybarek, Alicja Kazek-Kęsik

**Affiliations:** 1 Department of Physical Chemistry and Technology of Polymers, Faculty of Chemistry, Silesian University of Technology, Strzody 9 Str., 44-100 Gliwice, Poland; izabela.barszczewska-rybarek@polsl.pl; 2 Department of Inorganic Chemistry, Analytical Chemistry and Electrochemistry, Faculty of Chemistry, Silesian University of Technology, Krzywoustego 6 Str., 44-100 Gliwice, Poland; alicja.kazek-kesik@polsl.pl; 3 Biotechnology Centre, Silesian University of Technology, Krzywoustego 8 Str., 44-100 Gliwice, Poland

**Keywords:** urethane-dimethacrylates, quaternary ammonium compounds, photocurable copolymers, physicochemical properties, antibacterial activity

## Abstract

The growing scale of secondary caries and occurrence of antibiotic-resistant bacterial strains require the development of antibacterial dental composites. It can be achieved by the chemical introduction of quaternary ammonium dimethacrylates into dental composites. In this study, physicochemical and antibacterial properties of six novel copolymers consisting of 60 wt. % quaternary ammonium urethane-dimethacrylate analogues (QAUDMA) and 40 wt. % triethylene glycol dimethacrylate (TEGDMA) were investigated. Uncured compositions had suitable refractive index (*RI*), density (*d_m_*), and glass transition temperature (*Tg_m_*). Copolymers had low polymerization shrinkage (*S*), high degree of conversion (*DC*) and high glass transition temperature (*Tg_p_*). They also showed high antibacterial effectiveness against *S. aureus* and *E. coli* bacterial strains. It was manifested by the reduction in cell proliferation, decrease in the number of bacteria adhered on their surfaces, and presence of growth inhibition zones. It can be concluded that the copolymerization of bioactive QAUDMAs with TEGDMA provided copolymers with high antibacterial activity and rewarding physicochemical properties.

## 1. Introduction

The technology of dental materials was revolutionized due to the discovery of bisphenol A glycerolate dimethacrylate (Bis-GMA) by Bowen in 1962 [1]. From that moment, Bis-GMA, together with other dimethacrylate resins such as its ethoxylated derivative (Bis-EMA), urethane-dimethacrylate monomer (UDMA), and triethylene glycol dimethacrylate (TEGDMA), have become the most commonly used resins in restorative dentistry. The current literature shows that Bis-GMA, TEGDMA, UDMA, and Bis-EMA comprise, 75, 60, 52, and 21%, respectively, of all used composite dental materials [2]. This is explained by their satisfactory mechanical properties, suitable adhesion to enamel, and high esthetic properties [3,4,5]. Nevertheless, materials of that type have several drawbacks. They include relatively high polymerization shrinkage [6,7], which leads to the marginal gap formation. As it occurs between the filling surfaces and the adjacent tissues, it is a hospitable place for the bacteria accumulation process [8,9]. Another disadvantage of dimethacrylate-based composites is a higher tendency for bacteria accumulation on their surfaces when compared to enamel and other restorative materials, such as amalgams [9,10,11,12,13].

Bacteria of the *Streptococcus mutans* strain are the most detrimental for dental restorations and responsible for the secondary caries formation of all the oral bacteria [14,15,16,17]. They produce the lactic acid that damages teeth as well as restoration surfaces [9]. The *S. mutans* presence on the tooth/restoration surfaces facilitates the accumulation of other bacterial species. It is caused by the pH changes in the local environment [9]. Secondary caries are the main reason for failures of dimethacrylate-based dental restorations [18,19]. Compared to amalgams, this occurs one-and-a-half times more frequently [9,20].

The abovementioned drawbacks of dimethacrylate-based dental composites encouraged scientists to undertake the developmental work on the dimethacrylate systems of antibacterial activity. It is currently one of the main objectives of modern dental material engineering.

The utilization of methacrylate monomers with the quaternary ammonium groups (QAMs) as antibacterial components of the composite matrix seems to be the most suitable solution for future dental materials [21]. The methacrylate groups, present in their structure, have the ability to copolymerize with other monomers of the dimethacrylate matrix, resulting in one coherent chemically crosslinked macromolecule. It guarantees that the antibacterial agent, the quaternary ammonium moiety in this case, will not be released from the composite matrix, ensuring long-lasting antibacterial properties. At the same time, the covalent bonding of those monomers to the composite matrix provides stable physicochemical and mechanical material properties during the restoration lifetime.

In 1997, the first QAM—12-methacryloyloxydodecylpyridinium bromide (MDPB)—to have one methacrylate and one quaternary ammonium group was synthesized [22]. It showed satisfactory antibacterial activity against *S. mutans*, *Actinomyces viscosus*, and *Lactobacillus casei*, which was revealed as a consequence of its addition to a commercial dental primer [22] and dental adhesive [23]. That modification did not have any negative effects on the degree of conversion and bond strength to the enamel.

Satisfying results for MDPB led to the development of further mono-QAMs, which include a series of 2-dimethylaminoethyl methacrylate (DMAEMA) derivatives that varied in terms of the N-alkyl substituent length and counter ion type [24,25,26,27,28]. Their high antibacterial activity was also revealed by their addition to commercial dental materials. Apart from gaining antibacterial activity, the physicochemical and mechanical properties did not achieve the requirements for dental materials. For example, water sorption and water solubility increased [25], whereas hardness, flexural modulus and flexural strength decreased [25,26]. These serious drawbacks cannot be easily eliminated due to the fact that mono-QAMs decrease crosslink density in the composite matrix [29]. With just one methacrylate group the mono-QAM molecules work as pendant groups in the crosslinked polymer and the higher their content, the lower the crosslinking density [29]. This limits the concentrations of mono-QAM in the composite matrix to low values. The alternative to mono-QAMs which enables their above mentioned disadvantages to be overcome are dimethacrylates containing two dimethacrylate groups (di-QAM).

Di-QAMs known from the literature can be divided into two groups by the number of quaternary ammonium groups. The first class includes di-QAMs with one quaternary ammonium group such as the following: 2-methacryloxyethyl dodecyl methyl ammonium bromide (MAE-DB) [21,29,30], 2-methacryloxyethyl hexadecyl methyl ammonium bromide (MAE-HB) [21,29,30], bis(2-methacryloyloxyethyl)dimethylammonium bromide (IDMA-1) [31,32], quaternary ammonium urethane-dimethacrylates with cycloaliphatic cores (QUDMA) [33,34], and fully aliphatic quaternary ammonium urethane-dimethacrylates (IMQ) [35,36]. The next group includes di-QAMs with two quaternary ammonium groups such as the following: N,N′-bis [2-(methacryloxyloxy) ethyl] N,N,N′,N′ tetramethyl N,N′ butanediyl diammonium bromide (DMBB) [37], N,N′-bis[2-(methacryloxyloxy) ethyl] N,N,N′,N′ tetramethyl N,N′ hexanediyl diammonium bromide (DMBH) [37], and quaternized derivative of Bis-GMA (QABGMA) [38]. The above mentioned monomers were added to commercial dental materials to test their antibacterial properties. All of them expressed strong antibacterial activity against many bacteria strains, such as the following: *S. mutans* [21,29,30,31,32,33,34,37,38], *A. viscosus* [21], *Escherichia coli* [38], *Lactobacillus acidophilus* [21], *Streptococcus sanguinis* [21], *Porphyromonas gingivalis* [21], *Prevotella melaninogenica* [21], *Enterococcus faecalis* [21], *Pseudomonas aeruginosa* [38], *Staphylococcus aureus* [21,38], and *Bacillus subtillis* [38]. It was found that the antibacterial activity of di-QAMs depends on the number of quaternary nitrogen groups in their structures [39]. The di-QAMs with two quaternary ammonium groups offered better antibacterial activity at their lowest concentrations. Di-QAMs with one quaternary ammonium group were used in several percentages, whereas di-QAMs with two quaternary ammonium groups were used in a few percentages [37]. However, the physicochemical and mechanical properties of the modified materials were still insufficient [21,29,30,31,32,33,34,36,37,38].

**Figure 1:**
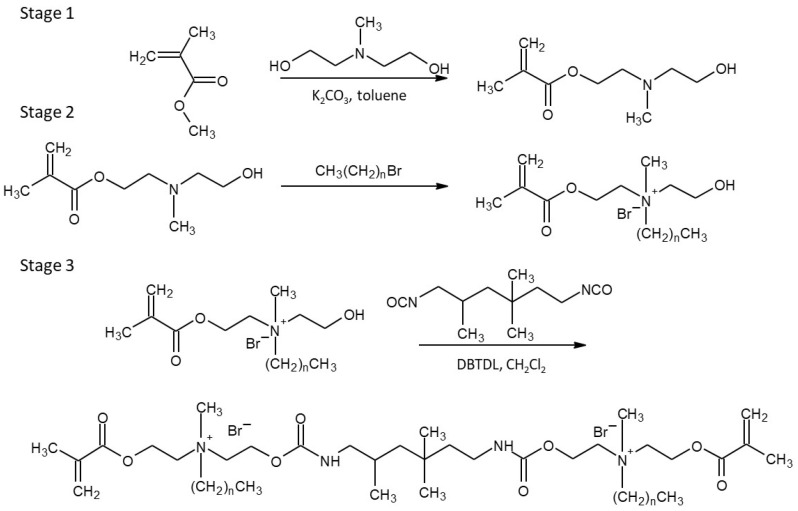
The synthesis route and chemical structure of the QAUDMA resins used in this study (*n* = 7–17). Stage 1—Synthesis of HAMA, Stage 2—Synthesis of QAHAMAS, Stage 3—Synthesis of QAUDMAs.

Although many QAMs described in the literature showed antibacterial properties, none of them allowed for the obtainment of a dental material with satisfactory physicomechanical characteristics. Therefore, our research area focuses on the development of innovative QAMs for dental applications. In our previous work [40], we successfully synthesized a family of six novel di-QAM resins, containing in their structure two quaternary ammonium groups (QAUDMA) substituted with the N-alkyl chain of varied length (Figure 1). They represent the UDMA analogues, as they have the trimethylhexamethylene diisocyanate (TMDI) core. Due to the two methacrylate groups, two quaternary ammonium groups, TMDI core and the lack of bisphenol A (BPA) moiety, we believe that these QAUDMAs could serve as valuable components of potential future bioactive dental materials [41]. Taking into account all these factors and the satisfying physicochemical properties of their liquid (resinous) forms, we qualified them for further studies, on testing the antibacterial and physicochemical properties of their crosslinked copolymers with common dental dimethacrylate.

The aim of this study was to verify that the presence of QAUDMA units in dimethacrylate copolymers provide them with antibacterial activity and satisfactory physicochemical properties. The research was carried on six dimethacrylate copolymers, consisting of QAUDMA 60 wt. % and TEGDMA 40 wt. %. Studies on liquid monomer compositions included the determination of refractive index (*RI*), density (*d_m_*), and glass transition temperature (*T_gm_*). Studies on copolymer physicochemical properties included the determination of density (*d_p_*), glass transition temperature (*T_gp_*), polymerization shrinkage (*S*), degree of conversion (*DC*), and water contact angle (*WCA*). Copolymer antibacterial activity was determined against *S. aureus* and *E. coli*.

## 2. Results

### 2.1. Properties of Liquid Monomer Compositions

In this work, seven dimethacrylate monomer formulations were prepared. Six of them contained QAUDMA monomers (QA:TEG), synthesized following the procedure described in the literature [40]. QAUDMA was mixed with TEGDMA in a weight ratio of 60 to 40%. One composition containing 60 wt. % Bis-GMA was prepared as a reference sample (BG:TEG).

The sample names and their compositions are specified in Table 1.

Liquid monomer compositions were tested for the refractive index (*RI*), density (*d_m_*), and glass transition temperature (*Tg_m_*). These results, along with the molecular weights (*MW*) and concentrations of double bonds (*x_DB_*), are summarized in Table 2.

The *RI* values of QA:TEG monomer compositions ranged from 1.4912 to 1.5001 and were lower than that of the BG:TEG reference sample (*RI* = 1.5129). The achieved results were usually statistically significantly different except for the QA10:TEG and QA12:TEG couple, which was statistically insignificantly different.

The *d_m_* values of the QA:TEG monomer compositions ranged from 1.07 to 1.15 g/cm^3^ and decreased with the increasing length of the N-alkyl substituent in QAUDMA. Compared to the BG:TEG reference sample (*d_m_* = 1.11 g/cm^3^), the *d_m_* of two compositions was higher: QA8:TEG (*d_m_* = 1.15 g/cm^3^) and QA10:TEG (*d_m_* = 1.13 g/cm^3^). However, these differences were statistically significant and statistically insignificant, respectively. The *d_m_* values of the remaining QA:TEG monomer compositions were lower than that of the BG:TEG reference sample and these differences were usually statistically significant, except for the QA12:TEG (*d_m_* = 1.11 g/cm^3^), which was not statistically significant.

**Figure 2:**
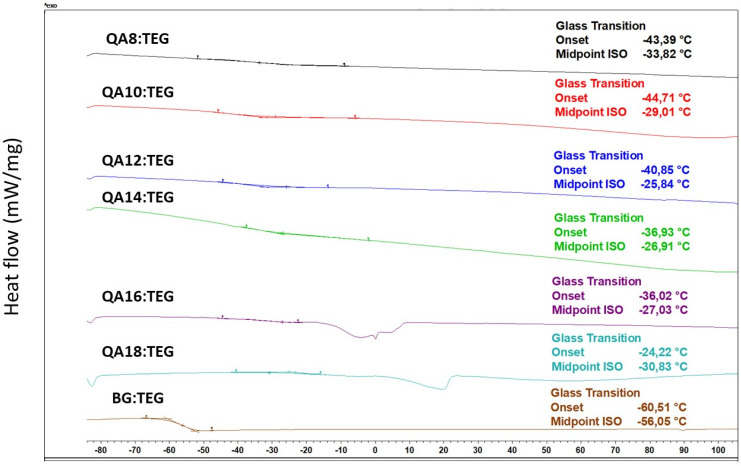
DSC thermograms of the liquid monomer compositions showing their glass transition temperatures.

The *Tg_m_* of QA:TEG monomer compositions ranged from −33.82 to −25.84 °C and first increased with the increasing length of the N-alkyl substituent in QAUDMA up to 12 carbon atoms and then decreased. These *Tg_m_* values were statistically significantly higher than that of the BG:TEG reference sample (*Tg_m_* = −56.05 °C). The DSC thermograms of liquid monomer compositions are shown in Figure 2.

### 2.2. Properties of Copolymers

Copolymers were obtained by photopolymerization under standard conditions for dental material curing. They were tested for physicochemical and antibacterial properties.

#### 2.2.1. Physicochemical Properties

In Table 3, the following physicochemical properties of copolymers are summarized: density (*d_p_*), experimental (*S_e_*) and theoretical (*S_t_*) polymerization shrinkages, degree of conversion (*DC*), and glass transition temperature (*Tg_p_*).

The *d_p_* values of QA:TEG copolymers ranged from 1.14 to 1.23 g/cm^3^ and decreased with the increasing length of the N-alkyl substituent in QAUDMA. The QA8:TEG (*d_p_* = 1.23 g/cm^3^) copolymer had a higher *d_p_* value than the BG:TEG reference sample (*d_p_* = 1.22 g/cm^3^), even though this difference was not statistically significant. The remaining QA:TEG copolymers were characterized by statistically significantly lower *d_p_* values than the BG:TEG reference sample.

The *S_t_* values of QA:TEG copolymers were lower than that of the BG:TEG reference sample (*S_t_* = 12.9%) and decreased with the increasing length of the N-alkyl substituent in QAUDMA and ranged from 9.0 to 10.4%. The *S_e_* values of QA:TEG copolymers ranged from 6.4 to 6.9%, and they were statistically significantly lower than that of the BG:TEG reference sample (*S_e_* = 8.4%).

**Figure 3:**
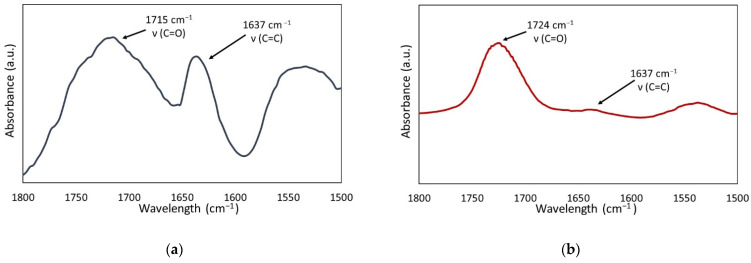
The representative FT IR spectra of the QA16:TEG: (**a**) liquid monomer composition and (**b**) copolymer.

The *DC* of the QA:TEG copolymers ranged from 84.0 to 88.7%. Its values initially increased with the lengthening of the N-alkyl substituent in QAUDMA from 8 to 14 carbon atoms. Further lengthening of the N-alkyl substituent in QAUDMA caused a slight decrease in *DC* values. The *DC* values of all QA:TEG copolymers were statistically significantly higher than that of the BG:TEG reference sample (*DC* = 64.8%). The representative FT IR spectra of the QA16:TEG liquid monomer composition and its copolymer are shown in Figure 3.

**Figure 4:**
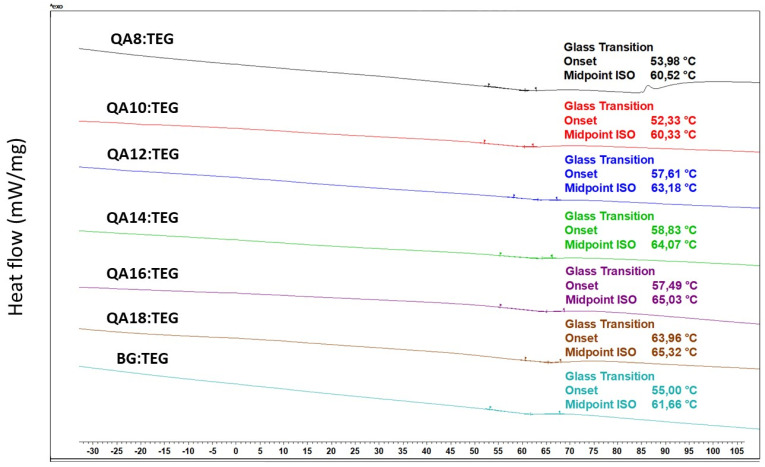
DSC thermograms of copolymers showing their glass transition temperatures.

The *Tg_p_* of the QA:TEG copolymers ranged from 60.33 to 66.32 °C and increased with the increasing length of the N-alkyl substituent in QAUDMA. The *Tg_p_* values of the QA8:TEG and QA10:TEG were statistically insignificantly lower than that of the BG:TEG reference sample (*Tg_p_* = 61.66 °C). The remaining QA:TEG copolymers usually had statistically significantly higher *Tg_p_* values than that of the BG:TEG reference sample, except for the QA12:TEG, which was statistically insignificantly higher. The DSC thermograms of copolymers are shown in Figure 4. The DSC thermograms also show the onset point values (*T_op_*), which ranged from 52.33 to 63.96 °C. The *T_op_* values of QA8:TEG and QA10:TEG copolymers were lower than that of the BG:TEG reference sample (*T_op_* = 55.00 °C). The remaining QA:TEG copolymers had higher *T_op_* values than that of the BG:TEG reference sample.

**Figure 5:**
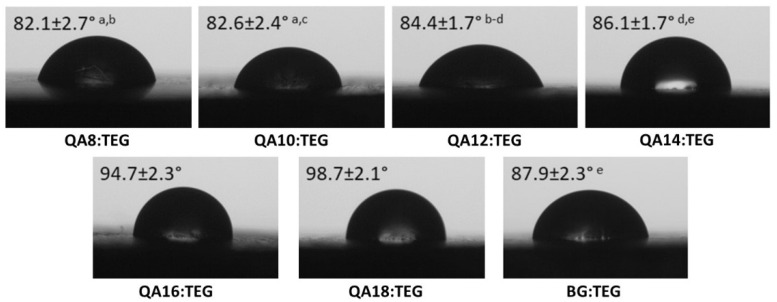
The images of deionized water droplets on the studied copolymer surfaces obtained from the goniometry camera. Lower case letters indicate statistically insignificant differences (*p* > 0.05) (non-parametric Wilcoxon test).

In Figure 5, the results for the water contact angle (*WCA*) are shown. The *WCA* values of QA:TEG copolymers ranged from 82.1 to 98.7° and increased with the increasing length of the N-alkyl substituent in QAUDMA. The *WCA* values of QA8:TEG to QA14:TEG copolymers were lower than the *WCA* value of the BG:TEG reference sample. These differences were usually statistically significant, except the difference for the QA14:TEG, which was statistically insignificant. The QA16:TEG and QA18:TEG copolymers were characterized by statistically significantly higher *WCA* values than the BG:TEG reference sample.

#### 2.2.2. Antibacterial Properties

**Figure 6:**
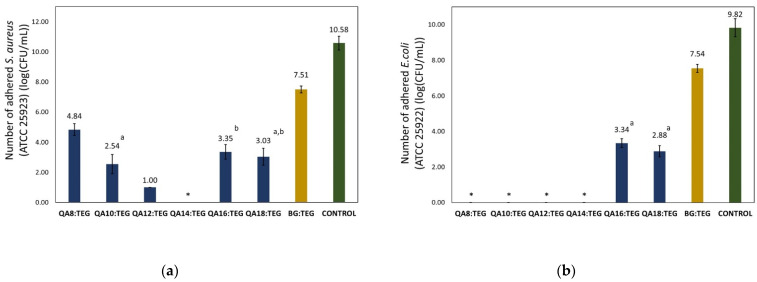
The results of the bacterial adhesion tests with (**a**) *S. aureus* (ATCC 25923) and (**b**) *E. coli* (ATCC 25922) on the studied copolymer surfaces after 18 h of incubation. An asterisk (*) indicates that no adhered bacteria were observed. Lower case letters indicate statistically insignificant differences (*p* > 0.05) (non-parametric Wilcoxon test).

In Figure 6, the results from the bacterial adhesion tests are shown. They were performed for two bacteria strains: *S. aureus* (ATCC 25923) and *E. coli* (ATCC 25922). All the QA:TEG copolymers showed statistically significant decreases in the number of adhered bacteria in comparison with the BG:TEG reference sample.

Initially, an increase in the N-alkyl substituent length from 8 to 14 carbon atoms resulted in a decrease in the number of adhered *S. aureus*. The maximum number of adhered bacteria was observed on the QA8:TEG copolymer surface, which was 4.84 log(CFU/mL). On the other hand, no adhered bacteria were observed on the QA14:TEG copolymer surface. The further increase in the N-alkyl substituent length of up to 18 carbon atoms caused an increase in the number of adhered bacteria.

No *E. coli* colonies were observed on the surfaces of QA8:TEG to QA14:TEG copolymers. The further increase in the N-alkyl substituent length caused colonization of bacteria on copolymer surfaces. The number of adhered bacteria on the QA16:TEG and QA18:TEG copolymer surfaces was 3.34 and 2.88 log(CFU/mL), respectively.

Table 4 shows the results for bacterial growth inhibition zones. The *S. aureus* growth inhibition zones were observed for all QA:TEG copolymers. They ranged from 6 to 19 mm and decreased with the increasing length of the N-alkyl substituent in QAUDMA. All these results were statistically significantly higher than that of the BG:TEG reference sample, except for the QA18:TEG, which was the same as the result for the BG:TEG reference sample.

The *E. coli* growth inhibition zones were only observed for copolymers from QA8:TEG to QA14:TEG. They ranged from 6 to 10 mm and decreased with the increasing length of the N-alkyl substituent in QAUDMA. All these results were statistically significantly higher than that of the BG:TEG reference sample, except for the QA14:TEG, which was statistically insignificantly higher than the result for the BG:TEG reference sample. QA16:TEG and QA18:TEG had no inhibition zones, the same as it was in the case of the BG:TEG sample.

**Figure 7:**
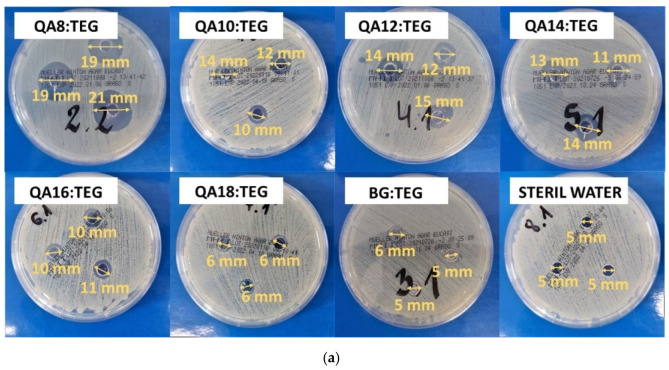

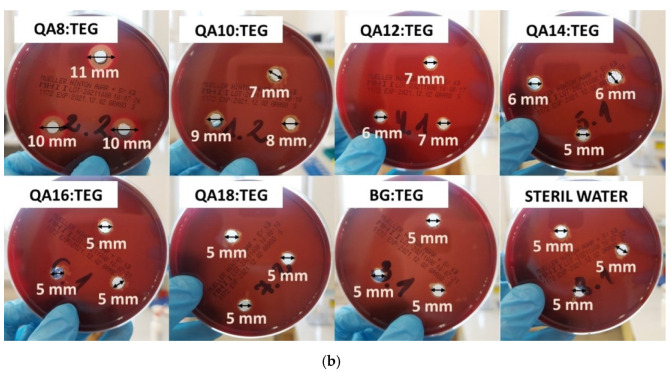
Images showing (**a**) *S. aureus* (ATCC 25923) and (**b**) *E. coli* (ATCC 25922) growth inhibition zones.

Growth inhibition zones of both tested bacteria strains are shown in Figure 7.

**Figure 8:**
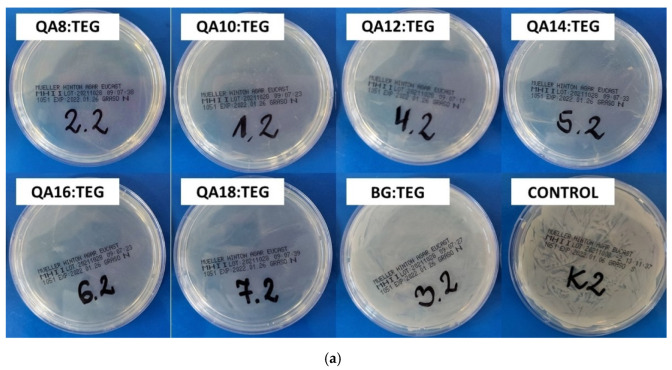

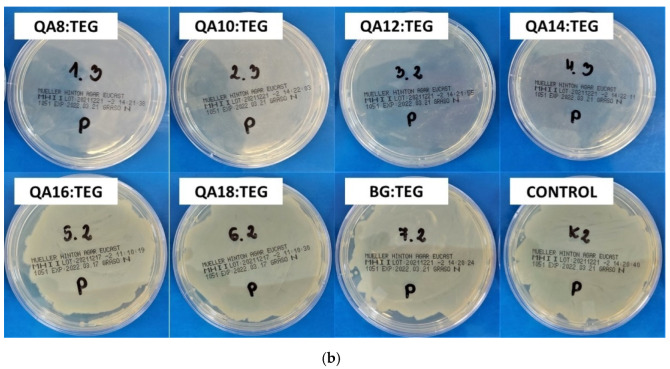
Images of the bacteria colonies (**a**) *S. aureus* (ATCC 25923) and (**b**) *E. coli* (ATCC 25922) that grew at the copolymer concentrations of 25 mg/mL.

Figure 8 shows the influence of studied copolymers on bacterial cell proliferation. The obtained results show that QA:TEG copolymers reduced the proliferation of *S. aureus* to a greater extent than of *E. coli*. All QA:TEG reduced the ability of *S. aureus* to proliferate at the concentration of 25 mg/mL. Only copolymers from QA8:TEG to QA14:TEG reduced the proliferation of *E. coli*, whereas QA16:TEG and QA18:TEG did not affect the proliferation of *E. coli* at this concentration. The BG:TEG reference sample did not affect the proliferation of both bacteria strains at this concentration.

## 3. Discussion

The global problem of the growing scale of dental caries brought the need for the development of dental composites with antibacterial properties. In response to the increasing number of antibiotic-resistant bacteria strains, the utilization of the dimethacrylate monomers with quaternary ammonium groups (QAMs) as bioactive components of composite matrices seems to be an interesting solution.

In our previous study [40], we synthesized and characterized six QAUDMA monomers, being the UDMA monomer analogues. They consisted of two methacrylate wings containing quaternary ammonium group substituted with an N-alkyl chain of various length, and the TMDI core. QAUDMAs were characterized by low polymerization shrinkage (*S*), high degree of conversion (*DC*)*,* and suitable refractive index (*RI*). Their great advantage was a liquid state; however, due to high viscosity, they cannot operate as standalone components of matrices in dental composites. Nevertheless, their physicochemical characteristics revealed that the QAUDMA monomers may serve as promising potential components of this type of material. Therefore, they were subjected to further examinations.

The aim of this study was to investigate the influence of the QAUDMA monomers on the physicochemical and antibacterial properties of their copolymers with triethylene glycol dimethacrylate (TEGDMA). For this purpose, a series of six dimethacrylate photocured copolymers containing 60 wt. % QAUDMAs and 40 wt. % TEGDMA were prepared. TEGDMA acted as a reactive diluent. A sample composed of 60 wt. % Bis-GMA and 40 wt. % TEGDMA served for comparison purposes.

Additionally, the physicochemical properties of liquid monomer compositions were also tested.

### 3.1. Properties of Liquid Monomer Compositions

The refractive index (*RI*) of QA:TEG liquid monomer compositions (Table 2) was within the range specified for dental materials, which should be from 1.46 to 1.55 [42]. Its average value was 1.33% lower than that of the BG:TEG reference sample. It indicates that the transparency of all QA:TEG liquid monomer compositions is similar to that of enamel [42].

The density of QA:TEG liquid monomer compositions (*d_m_*) decreased with the increasing length of the N-alkyl substituent in QAUDMA (Table 2). It indicates that the longer the N-alkyl substituent, the lower the molecular packing. It can be attributed to the increasing dimensions of the N-alkyl substituent after adopting the most energetically favorable conformation. However, the *d_m_* value of the BG:TEG reference sample was within the range of the *d_m_* values of the QA:TEG liquid monomer compositions. The lowest *d_m_* value was recorded for the QA18:TEG, which was 4.04% lower with respect to the BG:TEG reference sample. On the other hand, QA8:TEG was characterized by the highest *d_m_* value, which was 2.87% higher than that of the BG:TEG reference sample. Interestingly, the *d_m_* value of the QA8:TEG was almost the same as the *d_m_* value of the pure Bis-GMA monomer [40]. Regarding the fact that Bis-GMA has an aromatic core and two hydroxyl groups responsible for hydrogen bonding, the *d_m_* of the fully aliphatic QA8:TEG can be recognized as high.

The glass transition temperature (*Tg_m_*) values of all QA:TEG liquid monomer compositions were higher than that of the BG:TEG reference sample (Table 2). Initially, the *Tg_m_* values increased with the increasing length of the N-alkyl substituent up to 12 carbon atoms. Further extension of the N-alkyl substituent resulted in a reversed tendency. The lowest *Tg_m_* value was recorded for the QA8:TEG, which was higher by 22.25 °C than that of the BG:TEG reference sample. On the other hand, QA12:TEG was characterized by the highest *Tg_m_* value which was higher by 30.23 °C than that of the BG:TEG reference sample. As the *Tg_m_* value informs about the molecular mobility of monomers [43,44] it can be concluded that all QA:TEG liquid monomer compositions are characterized by lower mobility than the BG:TEG reference sample. Regarding the fact that the molecular mobility of the BG:TEG reference sample is restricted by the Bis-GMA structural properties, it can be concluded that strong molecular interactions occur between the monomers in tested QA:TEG liquid monomer compositions. They arise from the presence of strong hydrogen bonds between urethane groups [45]. This result coincides with the findings for *d_m_*.

### 3.2. Properties of Copolymers

#### 3.2.1. Physicochemical Properties

The density of copolymers (*d_p_*) was also determined (Table 3). Its values were greater than the corresponding *d_m_* values, due to polymerization.

As the polymerization of methacrylate groups causes volumetric contraction, the theoretical (*S_t_*) and experimental (*S_e_*) polymerization shrinkages were calculated (Table 3).

The polymerization shrinkage (*S*) is one of the most important parameters in the characteristics of dental materials. It is responsible for restoration stability from physicochemical, mechanical, and biological points of view. The lower the *S* value, the narrower the gap between the restoration and tooth tissue is. Therefore, the restoration stability is better and there is more limited space for bacteria colonization. From this perspective, low *S* values are desirable.

The *S_t_* values decreased as the N-alkyl substituent length increased, which is obvious as the QAUDMA molecular weight increased, and consequently the concentration of double bonds in QA:TEG decreased (Table 2). However, all of those values were lower than that of the BG:TEG reference sample. The highest *S_t_* value was calculated for the QA8:TEG, which was 19.24% lower than that of the BG:TEG reference sample. On the other hand, QA18:TEG was characterized by the lowest *S_t_* value, which was 29.87% lower than that of the BG:TEG reference sample.

The *S_e_* values of all QA:TEG copolymers were unaffected by the N-alkyl substituent length, which resulted in similar *S_e_* values, on average of 6.59%. Compared to the BG:TEG reference sample, the QA:TEG copolymers were characterized by lower *S_e_* values, on average by 21.32%. In comparison with the copolymers composed of 50 wt. % TEGDMA and 50 wt. % quaternary ammonium urethane-dimethacrylate with isophorne diisocyanate moieties in the molecule wings (UDMQA), all QA:TEG copolymers had lower S_e_ values. The S_e_ values of UDMQA-based copolymers ranged from 7.1 to 7.5% and were also unaffected by the N-alkyl substituent length [33].

The degree of conversion (*DC*) is another crucial parameter that needs to be taken into account while characterizing dental composites. The *DC* values of all QA:TEG copolymers were higher than that of the BG:TEG reference sample (Table 3). Moreover, those values can be recognized as very high as they were even higher than the *DC* in the TEGDMA homopolymer, being 84.2% [40]. The lowest *DC* value was recorded for the QA8:TEG, which was 29.82% higher than that of the BG:TEG reference sample. On the other hand, QA14:TEG was characterized by the highest *DC* value, which was 36.77% higher than that of the BG:TEG reference sample. This increase in the *DC* value with the increasing length of the N-alkyl substituent in QAUDMA suggests that the number of carbon atoms does not have a negative effect on the *DC*. It is probably that the N-alkyl chain adopts tangled conformations that take up little space. The presence of 16 and 18 carbon atoms in N-alkyl substituents caused meaningless decrease in the *DC* values. It implies that the longest N-alkyl substituents have tangled conformations, but they are more spacious. This conclusion coincides well with the results achieved for the *d_m_*. The same tendency was also observed for the UDMQA-based copolymers [33]. Their DC values ranged from 66.2 to 73.2%. UDMQA-based copolymers composed of UDMQAs having 12 and 14 carbon atoms in the N-alkyl substituents had lower DC than the copolymers composed of UDMQA monomers having 16 and 18 carbon atoms in N-alkyl substituents. Compared to them, all QA:TEG copolymers had significantly higher DC values. The lowest DC value among UDMQA copolymers observed for the UDMQA-14-based copolymer was 66.2%, whereas the lowest DC value among QA:TEG copolymers observed for the QA10:TEG copolymer was 84.0%.

The glass transition temperature of copolymers (*Tg_p_*) was determined as a basic physicochemical parameter that characterizes the stiffness of dental copolymers. A polymer used as a composite matrix must be in a glassy state at intraoral temperatures. This condition is met when the *Tg_p_* value is higher than the highest possible oral temperature [46] (for example, in most adults, an intraoral temperature above 37.2 °C is recognized as fever [47]). The *Tg_p_* of QA:TEG copolymers increased with the increasing length of the N-alkyl substituent in QAUDMA (Table 3). The QA8:TEG and QA10:TEG had similar *Tg_p_* values. The lowest *Tg_p_* value was recorded for the QA10:TEG, which was lower by 1.33 °C than that of the BG:TEG reference sample. On the other hand, QA18:TEG was characterized by the highest *Tg_p_* value, which was higher by 4.66 °C than that of the BG:TEG reference sample. It can be concluded that the results achieved for *Tg_p_* guarantee high mechanical stability of the tested copolymers with temperature changes in the oral cavity. The pattern of *Tg_p_* changes is similar to that observed for the *Tg_m_*. This suggests that the molecular stiffness of the copolymer is not adversely affected by the N-alkyl substituent length. Despite its increase, probably due to the highly tangled conformation, it did not show a plasticizing effect on the copolymer network. On this basis, it can be concluded that the strong intermolecular interactions play a major role in *Tg_p_*, mainly due to the presence of urethane groups, which form one of the strongest hydrogen bonds [45].

The water contact angle (*WCA*) was tested in order to estimate the hydrophobic nature of the copolymer surfaces. The *WCA* values of the QA:TEG copolymers increased with the increasing length of the N-alkyl substituent (Figure 5). It means that the hydrophilic nature of these surfaces decreased [48]. The obtained results are interesting because the behavior of the tested surfaces nature was unpredictable. On one side, two quaternary nitrogen atoms, which are present in the QAUDMA structure, provide hydrophilicity. On the other side, the N-alkyl substituent is highly hydrophobic. The surfaces of the copolymers from QA8:TEG to QA14:TEG were hydrophilic because their *WCA* values were lower than 90°. The surfaces of the QA16:TEG and QA18:TEG copolymers can be recognized as hydrophobic because their *WCA* values were higher than 90° [49]. It suggests that the presence of N-alkyl substituents with 16 and 18 carbon atoms turns the surface nature of studied copolymers from hydrophilic to hydrophobic. The lowest *WCA* value was recorded for the QA8:TEG, which was 6.60% lower than that of the BG:TEG reference sample. On the other hand, QA18:TEG was characterized by the highest *WCA* value, which was 12.31% higher than that of the BG:TEG reference sample.

#### 3.2.2. Antibacterial Properties

Quaternary ammonium compounds (QACs) are known for their high antibacterial activity. It results from the presence of positively charged nitrogen atoms, which can electrostatically interact with the negatively charged surface of bacteria cells. This causes the release of potassium ions and other cytoplasmic components relevant for the proper functioning of the bacteria. This leads to an increase in the osmotic pressure inside the bacteria cell and consequently to its lysis [50,51].

The antibacterial activity of QA:TEG copolymers was determined against Gram-positive (*S. aureus* ATCC 25923) as well as Gram-negative (*E. coli* ATCC 25922) bacteria.

The number of bacteria from both strains that adhered onto the QA:TEG copolymer surfaces was lower than that on the BG:TEG reference sample (Figure 6). The number of *S. aureus* that adhered to the QA:TEG copolymers initially decreased with the increasing length of the N-alkyl substituent in QAUDMA from 8 to 14 carbon atoms. Further lengthening of the N-alkyl substituent resulted in an increase in the number of adhered *S. aureus*. The spectacular result was observed for the QA14:TEG copolymer on which surface no adhered bacteria were observed. A satisfactory result was also achieved for the QA12:TEG copolymer, for which the number of adhered *S. aureus* decreased by 86.68% in comparison with the BG:TEG reference sample. The remaining decreases in the number of adhered *S. aureus* were lower, from 35.57 to 66.18%, in comparison with the BG:TEG reference sample. *E. coli* were only observed on the surfaces of the QA16:TEG and QA18:TEG copolymers. The percentage difference between the number of *E. coli* adhered on their surfaces and the BG:TEG reference sample surface was 55.70 and 61.80%, respectively. No *E. coli* were observed on the surfaces of the remaining QA:TEG copolymers (from QA8:TEG to QA14:TEG). The pattern observed for the changes in bacteria number with the changing length of the N-alkyl substituent is consistent with the literature data. The N-alkyl substituent length has a crucial impact on the antibacterial properties of QACs. If the N-alkyl substituent length is lower than the length of the lipid constituting the bacteria cell wall the interactions between the N-alkyl substituent and the lipid chain are weaker. When the length of the N-alkyl substituent is comparable to the length of the lipid chain, the interactions between them increase, and therefore the antimicrobial properties of QACs increase, because the greater surface of the bacteria cell wall is destabilized [52].

The bacterial growth inhibition zones were tested too. The bacterial growth inhibition zones decreased with the increasing length of the N-alkyl substituent in QAUDMA for both bacteria strains (Table 4 and Figure 7). In the case of *S. aureus*, QA8:TEG copolymer revealed the highest inhibition zone, which was of 19 mm. The lowest inhibition zone of 6 mm was recorded for the QA18:TEG copolymer, which was the same as that for the BG:TEG reference sample. In the case of *E. coli*, QA8:TEG copolymer revealed the highest inhibition zone, which was 10 mm. The lowest inhibition zones of 6 mm were recorded for the QA14:TEG copolymer. The QA16:TEG and QA18:TEG copolymers, did not reveal the inhibition zone, the same as the BG:TEG reference sample. The observations for bacterial growth inhibition zones can be explained by the complex effect of the monomer *MW*, polymer *DC* and *WCA*. As the *MW* and *DC* increased with the increasing length of the N-alkyl substituent in QAUDMA, the amount of sol fraction that can leach from the QA:TEG copolymers with shorter N-alkyl substituent in QAUDMA will probably be higher than that from the QA:TEG copolymers with longer N-alkyl substituent in QAUDMA. The same relationship was observed for the *WCA*, which means that the hydrophobicity of the QA:TEG copolymers increased with the increasing length of the N-alkyl substituent in QAUDMA. It is obvious that the higher the QA:TEG copolymer hydrophobicity, the lower its compatibility with water resulting in lower monomer leaching. Significant difference in the inhibition zone between QA8:TEG and QA10:TEG copolymers indicates that the monomer *MW* might play major role. It is known from the literature that monomers of lower *MW* have greater ease to migrate from the polymer structure in aqueous environment [53]. As QA8:TEG liquid monomer composition has the lowest *MW*, it is likely that the amount of monomer that will leach out of the copolymer structure will be high, and same the concentration of bioactive quaternary ammonium groups observed in the monomer leachable fraction will be the highest.

The influence of QA:TEG copolymers on the bacteria cell proliferation was determined too. The obtained results showed that the QA:TEG copolymers reduced the proliferation of *S. aureus* more efficiently than *E. coli* (Figure 8). All QA:TEG copolymers reduced the proliferation of *S. aureus*, whereas only copolymers from QA8:TEG to QA14:TEG reduced the proliferation of *E. coli*. QA16:TEG and QA18:TEG copolymer did not affect the proliferation of *E. coli*, the same as it was in the case of the BG:TEG reference sample. The latter one also did not affect the proliferation of *S. aureus*.

## 4. Materials and Methods

### 4.1. Chemicals and Reagents

Bis-GMA (bisphenol A glycerolate dimethacrylate, Sigma Aldrich, St. Louis, MO, USA), 1-bromoocatne (Acros Organics, Geel, Belgium), 1-bromododecane (Acros Organics, Geel, Belgium), 1-bromododecane (Acros Organics, Geel, Belgium), 1-bromotetradecane (Acros Organics, Geel, Belgium), 1-bromohexadecane (Acros Organics, Geel, Belgium), 1-bromooctadecane (Acros Organics, Geel, Belgium), CQ (camphorquinone, Sigma-Aldrich, St. Louis, MO, USA), chloroform (POCH S.A., Gliwice, Poland), DBTDL (dibutyltin dilaurate, Fluka, Charlotte, NC, USA), DMAEMA (2-dimethylaminoethyl methacrylate, Sigma Aldrich, St. Louis, MO, USA), methylene chloride (POCH S.A., Gliwice, Poland), potassium carbonate (POCH S.A., Gliwice, Poland), MDEA (N-methyldiethanolamine, Acros Organics, Geel, Belgium) MMA (methyl methacrylate, Acros Organics, Geel, Belgium), phenotiazine (PTZ, Sigma Aldrich, St. Louis, MO, USA), potassium bromide (Sigma Aldrich, St. Louis, MO, USA), potassium carbonate (POCH S.A., Gliwice, Poland), TEGDMA (triethylene glycol dimethacrylate, Sigma Aldrich, St. Louis, MO, USA), TMS (tetramethylsilane Sigma-Aldrich, St. Louis, MO, USA), toluene (POCH S.A., Gliwice, Poland), and trimethylhexamethylene diisocyanate (TMDI, Tokyo Chemical Industry, Tokyo, Japan) were used as received. TSB (tryptic soy broth, Biomaxima, Poland) was dissolved in sterile water in accordance with the information posted on the packaging.

### 4.2. Monomers Synthesis

QAUDMAs were synthesized following the procedure described in the literature [40]. An amount of 1 mol (100.12 g) of MMA, 0.67 mol (79.85 g) of MDEA, K_2_CO_3_ (reaction catalyst, 8 wt. %), PTZ (polymerization inhibitor, 500 ppm), and toluene (400 mL) were placed in a 1000 mL round-bottom flask equipped with a laboratory distillation kit. The system was heated to boil until the temperature on the top of the column increased from 65 to 100 °C. The reaction was stopped after 2.5 h and K_2_CO_3_ was filtered. The filtrate was washed twice with distilled water. Water fraction was then washed three times with chloroform. Chloroform was removed on a rotary evaporator under 30 mbar and the crude product was achieved (HAMA). HAMA was distilled under 7 mbar.

In the next step, 0.107 mol (20.0 g) of HAMA, alkyl bromide (0.107 mol, weight depending on the alkyl bromide), PTZ (500 ppm) were placed in a 250 mL round-bottom flask equipped with a standard reaction kit. The reaction was heated to 82 °C from 82 to 168 h (depending on the alkyl bromide) resulting in QAHAMA.

In the final step, QAHAMA was reacted with 0.054 mol (11.24 g) of TMDI in the presence of DBTDL (reaction catalyst, 0.03 wt. %) and PTZ (500 ppm) in 50 wt. % solution in methylene chloride. The reaction mixture was heated to boil (42 °C) and kept for 4 h at this temperature. Then, methylene chloride was removed on a rotary evaporator under 7 mbar.

The NMR spectra of the QAUDMA monomers are shown in our previous publication [40], which describes the synthesis and physicochemical properties of QAUDMA monomers.

### 4.3. Sample Preparation

Seven dimethacrylate formulations were obtained. Six of them consisted of 60 wt. % QAUDMAs and 40 wt. % TEGDMA. For comparison purposes, one formulation containing 60 wt. % Bis-GMA and 40 wt. % TEGDMA was also prepared. Mixing was performed by mechanical stirring at 60 °C. Later, the initiating system (0.4 wt. % CQ—initiator, 1 wt. % DMAEMA—activator) was added. Final formulations were used to fill the following molds:

1. Square-shaped glass molds (90 mm × 90 mm × 4 mm (length × width × thickness));
2. Disc-like Teflon molds (15 mm × 1.5 mm (diameter × thickness));
3. Disc-like Teflon molds (10 mm × 3 mm (diameter × thickness)).

Formulations were then photopolymerized with the use of a UV-VIS lamp (280–780 nm wavelength, radiation exitance: 2400 mW/cm^2^, Ultra Vitalux 300, Osram, Munich, Germany) for 1 h, at room temperature. Before irradiation compositions were covered with PET film to reduce oxygen inhibition.

### 4.4. Physicochemical Properties

#### 4.4.1. Degree of Conversion

The degree of conversion (*DC*) was determined by the Fourier-transform infrared spectroscopy (FT-IR) using Spectrum Two (Perkin-Elmer, Waltham, MA, USA) spectrometer. The liquid monomer compositions were tested in a form of thin film placed between two KBr pellets. The copolymers were powdered, sieved to a grain size less than 25 µm, mixed with KBr, and formed into pellets. Examinations were performed with 128 scans at a resolution of 1 cm^−1^.

The DC was calculated according to Equation (1):

(1) $$DC\;\left(\%\right)=\left(1-\frac{{\left(\frac{A_{C=C}}{A_{C=O}}\right)}_{copolymer}}{{\left(\frac{A_{C=C}}{A_{C=O}}\right)}_{liquid\;monomer\;composition}}\right)\times 100,$$

where the following are defined:

*A_C_=_C_*—the absorption intensity of the band, corresponding to the carbon-carbon double bond stretching vibrations, at 1637 cm^−1^,
*A_C_=_O_*—the absorption intensity of the band, corresponding to the carbonyl group stretching vibrations, at 1715 cm^−1^.

#### 4.4.2. Glass Transition Temperature

Glass transition temperature (*T_g_*) was determined for liquid monomer compositions and their copolymers, utilizing Differential Scanning Calorimeter DSC 3 (Mettler Toledo, Greifensee, Switzerland) in agreement with the ISO 11357-2:2020 standard [54]. All measurements were performed in standard aluminum crucibles (2.5 mg sample weight) with a heating rate of 10 K/min, within the temperature range from −90 to 200 °C, in the air. The *T_g_* was taken as the midpoint of the transition region.

#### 4.4.3. Density and Polymerization Shrinkage

The density of liquid monomer compositions (*d_m_*) was determined utilizing a 1 mL pyknometer in agreement with the ISO 1675 standard [55]. The density of copolymers (*d_p_*) was determined according to the methodology based on Archimedes’ principle, utilizing analytical balance (XP Balance, Mettler Toledo, Greifensee, Switzerland) equipped with a density determination kit.

The experimental polymerization shrinkage (*S*) was calculated according to Equation (2):

(2) $$S\;\left(\%\right)=\left(1-\frac{d_{m}}{d_{p}}\right)\times 100,$$

where the following are defined:

*d_m_*—the liquid monomer composition density,
*d_p_—*the copolymer density.

The theoretical polymerization shrinkage (*S_t_*) was calculated according to Equation (3):

(3) $$S_{t}\;\left(\%\right)=\left(\frac{f\times ∆V\times d_{m}}{MW}\right)\times 100,$$

where the following are defined:

*MW*—the molecular weight of liquid monomer composition,
*d_m_*—the liquid monomer composition density,
Δ*V*—the decrease in molar volume of one methacrylate group due to its polymerization (Δ*V* = 22.5 cm^3^/mol [3]),
*f*—the number of methacrylate groups in the monomer molecule (*f* = 2).

#### 4.4.4. Refractive Index

The refractive index (RI) was determined in agreement with the ISO 489:1999 standard [56]. An amount of 2 mL of liquid monomer compositions was placed on DR 6100T (Krüss Optronic, Hamburg, Germany) a refractometer plate, and the measurement was carried out at 20 °C.

#### 4.4.5. Water Contact Angle

The water contact angle (*WCA*) was determined using the sessile drop method utilizing the OCA 15EC (Data Physics, Filderstadt, Germany) goniometer. Rectangular copolymer samples of 40 mm × 20 mm × 4 mm (length × width × thickness) were sanded with fine sanding paper. Then, 4 µL of deionized water were dropped on the sample surface.

### 4.5. Antibacterial Properties

The antibacterial tests were carried out using reference *S. aureus* (ATCC 25923) and *E. coli* (ATCC 25922) bacteria strains. Before analysis, the bacteria were cultured in TSB culture medium at 37 °C for 18 h (incubator POL-EKO, Wodzisław Śląski, Poland).

#### 4.5.1. Bacterial Adhesion

The bacterial adhesion tests were performed on disc-like copolymer samples of 10 mm × 3 mm (diameter × thickness), which were sanded with fine sanding paper.

Specimens were placed in 25 mL test tubes, immersed in 1 mL of bacterial suspension (~5 × 10^6^ CFU/mL), and incubated at 37 °C for 18 h. Then, the bacterial suspension was removed from the test tubes, and samples were washed slightly with sterile water. The washed specimens were placed in clean test tubes, immersed in 1 mL of sterile water, and vortexed for 1 min at 3000 rpm to remove the adhered bacteria from their surfaces. Subsequently, 100 µL of the achieved bacteria suspensions were mixed with 0.9% NaCl at concentrations of 1:10, 1:100, 1:1000, 1:10,000, 1:100,000, and 1:1,000,000. An amount of 100 µL of those solutions was spread on agar plates (Müller-Hinton agar, Diag-Med, Warsaw, Poland) and incubated at 37 °C for 18 h. Thereafter, the bacterial colonies were counted.

#### 4.5.2. Inhibition Zone

The bacterial growth inhibition zones were measured utilizing disc-like copolymer samples of 10 mm × 3 mm (diameter × thickness), which were sanded with fine sanding paper. They were immersed in 1 mL of sterile water and stored for seven days at room temperature. Then, samples were removed from the water and the obtained solutions were used for further analysis.

An amount of 100 µL of the bacterial suspension (~5 × 10^8^ CFU/mL) was spread onto agar plates. Then, three holes of 5 mm in diameter were cut from each agar plate (Müller-Hinton agar, Diag-Med, Warsaw, Poland). Next, 100 µL of the previously prepared sample solutions was placed into each hole. The agar plates were incubated at 37 °C for 24 h. Thereafter, the bacterial growth inhibition zones were measured.

#### 4.5.3. Bacterial Cell Proliferation

The influence of copolymers on bacterial cell proliferation was determined utilizing powdered copolymer specimens of a grain size smaller than 25 µm.

A quantity of 50 mg of powdered samples was placed in glass test tubes, immersed in 2 mL of TSB solution, and vortexed for 1 min at 6000 rpm to obtain homogenous suspensions of powdered copolymers in TSB. Then, 20 µL of bacterial suspension (~5 × 10^8^ CFU/mL) were added, vortexed for 10 s at 2000 rpm, and incubated at 37 °C for 18 h. Thereafter, specimens were vortexed again for 10 s at 2000 rpm, and then 100 µL of suspensions was spread on agar plates. The agar plates were incubated at 37 °C for 18 h. Finally, the bacterial colonies were counted.

### 4.6. Statistical Analysis

Each experiment was repeated for five independent samples. The results were expressed as an average value and corresponding standard deviation (*SD*). Statistical analysis was performed with the Statistica 13.1 (TIBCO Software Inc., Palo Alto, CA, USA) software. The statistical significance of the results was determined by the non-parametric Wilcoxon test with a significance level (*p*) of 0.05.

## 5. Conclusions

A series of QA:TEG dimethacrylate copolymers consisting of 60 wt. % QAUDMA as a bioactive component and 40 wt. % TEGDMA as a reactive diluent have high antibacterial activity and rewarding physicochemical properties. Liquid QA:TEG monomer compositions showed a suitable refractive index and glass transition temperature. QA:TEG copolymers were characterized by low polymerization shrinkage, a high degree of conversion, and high glass transition temperature. High antibacterial effectiveness against *S. aureus* and *E. coli* of QA:TEG copolymers was manifested by the reduction in cell proliferation, a decrease in the number of bacteria adhered on their surfaces, and the presence of growth inhibition zone. The majority of the tested properties changed depending on the length of the N-alkyl substituent in QAUDMAs. The following were observed (i) *d_m_* and *d_p_* values decreased with the lengthening of N-alkyl substituent; (ii) *Tg_m_* values initially increased with the lengthening of N-alkyl substituent up to 12 carbon atoms and then decreased; (iii) *Tg_p_* values increased with the lengthening of N-alkyl substituent; (iv) *S_t_* values decreased with the lengthening of N-alkyl substituent, whereas the *S_e_* values were unaffected by the length of the N-alkyl substituent; (v) *DC* values were unaffected by the length of the N-alkyl substituent; (vi) hydrophobicity of QA:TEG copolymers increased with the lengthening of N-alkyl substituent; (vii) the number of bacteria that adhered onto copolymer surfaces initially decreased with the lengthening of N-alkyl substituent up to 14 carbon atoms and then increased; (viii) inhibition zones decreased with the lengthening of N-alkyl substituent. Taking into account all tested properties, it can be seen that the copolymers composed of the QAUDMA monomers with 10, 12, and 14 carbon atoms in N-alkyl substituent offer the physicochemical and antibacterial properties that are the most suitable for dental materials. Therefore, it is worth to get the QAUDMA copolymers to further research towards the achievement of antibacterial dimethacrylate dental materials.

## Figures and Tables

**Table 1:** The sample names and compositions. The QAUDMA monomers are abbreviated to QA, accompanied by a number which represents the number of carbon atoms in the N-alkyl substituent. Bis-GMA is abbreviated as BG, and TEGDMA is abbreviated as TEG.

Sample Name		Composition							
		QA8	QA10	QA12	QA14	QA16	QA18	BG	TEG
QA8:TEG	wt. %	60							40
	mol%	30.7							69.3
QA10:TEG	wt. %		60						40
	mol%		29.5						70.5
QA12:TEG	wt. %			60					40
	mol%			28.4					71.6
QA14:TEG	wt. %				60				40
	mol%				27.4				72.6
QA16:TEG	wt. %					60			40
	mol%					26.4			73.6
QA18:TEG	wt. %						60		40
	mol%						25.5		74.5
BG:TEG	wt. %							60	40
	mol%							45.6	54.4

**Table 2:** Properties of studied liquid monomer compositions: molecular weight (*MW*), the concentration of double bonds (*x_DB_*), refractive index (*RI*), density (*d_m_*), and glass transition temperature (*Tg_m_*). Lower case letters indicate statistically insignificant differences (*p* > 0.05) with a column (non-parametric Wilcoxon test).

Sample Name	*MW* (g/mol)	*x_DB_* (mol/kg)	*RI* ^1^	*d_m_* (g/cm^3^)		*Tg_m_* (°C)	
				Average	SD	Average	SD
QA8:TEG	496	4.03	1.5001	1.15	0.00	−33.82 ^a^	1.34
QA10:TEG	505	3.96	1.4914 ^a^	1.13 ^a^	0.00	−29.01 ^b–e^	2.13
QA12:TEG	512	3.90	1.4912 ^a^	1.11 ^b,c^	0.01	−25.84 ^b,f–h^	3.12
QA14:TEG	520	3.85	1.4932	1.09 ^d^	0.01	−26.91 ^c,f,i^	3.06
QA16:TEG	526	3.80	1.4895	1.08 ^b,d,e^	0.01	−27.03 ^d,g,i,j^	1.36
QA18:TEG	533	3.75	1.4919	1.07 ^e^	0.01	−30.83 ^a,e,h,j^	3.73
BG:TEG	389	5.14	1.5129	1.11 ^a,c^	0.01	−56.05	5.87

^1^ Standard deviation for the *RI* values was 0.0001 in every case.

**Table 3:** Physicochemical properties of studied copolymers: density (*d_p_*), theoretical polymerization shrinkage (*S_t_*), experimental polymerization shrinkage (*S_e_*), degree of conversion (*DC*) and glass transition temperature (*Tg_p_*). Lower case letters indicate statistically significant differences (*p* ≤ 0.05) with a column (non-parametric Wilcoxon test).

Sample Name	*d_p_* (g/cm^3^)		*S_t_* (%)	*S_e_* (%)		*DC* (%)		*Tg_p_* (°C)	
	Average	SD		Average	SD	Average	SD	Average	SD
QA8:TEG	1.23 ^a–e^	0.01	10.4	6.6 ^a^	0.3	84.2 ^a–f^	1.2	60.52 ^a–d^	0.79
QA10:TEG	1.20 ^a,f–i^	0.00	10.0	6.4 ^b^	0.3	84.0 ^a,g–k^	0.9	60.33 ^e–h^	1.37
QA12:TEG	1.18 ^b,f,j–l^	0.01	9.7	6.5 ^c^	0.6	86.0 ^b,g,l,m^	1.2	63.18 ^a,e,i^	1.43
QA14:TEG	1.17 ^c,g,m–o^	0.00	9.4	6.9 ^d^	0.6	88.7 ^c,h,n^	1.4	64.07 ^b,f,j,k^	1.15
QA16:TEG	1.16 ^d,h,j,m,p,q^	0.00	9.2	6.5 ^e^	1.0	87.1 ^d,i,l,o^	1.1	65.03 ^c,g,l^	0.73
QA18:TEG	1.14 ^e,i,k,n,p,r^	0.01	9.0	6.5 ^f^	0.9	87.1 ^e,j,p^	0.9	66.32 ^d,h–j,m^	1.23
BG:TEG	1.22 ^f,l,o,q,r^	0.01	12.9	8.4 ^a–f^	0.6	64.8 ^f,k,m–p^	1.6	61.66 ^k–m^	0.58

**Table 4:** *S. aureus* (ATCC 25923) and *E. coli* (ATCC 25922) growth inhibition zones. The value of 5 mm indicates that no inhibition zone was observed. Lower case letters indicate statistically insignificant differences (*p* > 0.05) with a column (non-parametric Wilcoxon test).

Sample Name	Inhibition Zone (mm)			
	*S. aureus* (ATCC 25923)		*E. coli* (ATCC 25922)	
	Average	SD	Average	SD
QA8:TEG	19	1	10	1
QA10:TEG	12 ^a,b^	1	8	1
QA12:TEG	13 ^a,c^	2	7 ^a^	1
QA14:TEG	13 ^b,c^	2	6 ^a–e^	1
QA16:TEG	10	1	5 ^b,f–h^	0
QA18:TEG	6 ^d,e^	1	5 ^c,f,i,j^	0
BG:TEG	6 ^d,f^	1	5 ^d,g,i,k^	0
STERILE WATER	5 ^e,f^	0	5 ^e,h,j,k^	0

## Data Availability

Data supporting reported results are available from the authors.
